# In search of common ground - nephrologists’ experiences in preparing and informing patients on the path to end-stage kidney disease

**DOI:** 10.1186/s12882-025-04023-4

**Published:** 2025-02-28

**Authors:** Jenny Lindberg, Mats Johansson, Linus Broström

**Affiliations:** 1 Department of Clinical Sciences Lund, Medical Ethics, Lund University, BMC I12, Box 117, Lund, 22100 Sweden; 2 Department of Nephrology, Skåne University Hospital, Malmö, Sweden

**Keywords:** End-stage kidney disease, Dialysis, Conservative kidney management, Patient information, Shared decision-making

## Abstract

**Background:**

Patient education and dialogue are important when choosing a future treatment strategy for patients with chronic kidney disease. To support patients in their decision-making process, it is critical to provide information in a way that patients can understand. This study was conducted to understand how nephrologists view the goals of information sharing, the challenges involved, and the strategies used as part of treatment planning.

**Methods:**

This study had a qualitative design using semi-structured interviews with 14 practicing nephrologists working in different hospitals in Sweden and with experience in providing information to patients approaching the need for dialysis. The interviews were conducted in 2022. The data were analyzed using qualitative content analysis.

**Results:**

The results are presented thematically under the headings Objectives, Content, Challenges, and Strategies. Participants tried to find common ground with patients, in terms of shared knowledge, shared views on the appropriate decision-making process, and ultimately also agreement on which treatment option was best. There was a tension between allowing patients to make their own decisions and guiding patients to make decisions with the best outcomes as judged by the nephrologist. Achieving common ground was not always possible, both because of factors related to the patient’s preferences or limited capacity, and because of boundaries set by the physician to protect the patient from unwarranted or harmful information. Dealing with competing sources of information was seen as challenging. The nephrologists felt a professional responsibility for their treatment recommendations, combined with uncertainty about which patient would benefit from dialysis and when to start.

**Conclusions:**

Planning future treatment for patients with chronic kidney disease involves a complex information process that leaves room for both paternalism and respect for autonomy. Nephrologists face many competing challenges when discussing treatment options with patients. These challenges should be taken into account in the development of support for nephrologists in the area of information sharing.

## Background

Patients with progressive chronic kidney disease who are not candidates for kidney transplantation will eventually face the choice of starting dialysis to replace lost kidney function or opting for conservative kidney management. This situation is quite common. In Sweden, the number of patients on dialysis was 4082 in 2023 [1]. Of these patients, the majority (3052) were treated in hospital dialysis units, while 924 received peritoneal dialysis and 106 had home hemodialysis [1].

Dialysis is a time-consuming treatment that does not always alleviate all the symptoms that patients experience. Over time, there is often a progression of symptoms and underlying medical conditions, both somatic and mental, requiring more hospital care [2–5]. Committing to a treatment plan is typically a high-stakes decision, involving several important considerations. Although nephrologists are the ones who present treatment options, it is the patients who ultimately give or withhold consent. Patients therefore need adequate information about the disease, the available treatment options and their likely outcomes, delivered in an understandable and otherwise appropriate manner. Nephrologists are central to the provision of such information. Their perspectives on the goals of information sharing, what should be shared, the challenges they face, and the strategies they employ to achieve these goals and overcome the challenges, can have a significant impact on what information reaches the patient and how it will be perceived.

To explore the prospects for successful physician-patient dialogue in the face of potentially difficult treatment decisions, it is important to learn about nephrologists’ perspectives on sharing information with patients. Information sharing in the context of progressive kidney disease has been studied previously, but mainly from the specific perspective of the prospects for implementing shared decision-making, empowering patients to participate more fully in decision making and tailoring treatment to their personal needs and preferences [6–9]. This article addresses the complexities of information sharing more broadly, by exploring the attitudes and experiences of Swedish nephrologists regarding the process of informing patients considering whether to start dialysis, as well as the modality of dialysis chosen - either self-managed home dialysis or staff-managed hospital dialysis.

## Methods

### Design

The study had a qualitative design using semi-structured interviews with practicing nephrologists.

### Procedure and participants

Nephrologists with experience of informing patients before starting long-term dialysis were invited to participate in the study via the mailing list of the Swedish Society of Nephrology member registry. 14 nephrologists (participants) were included consecutively. The participants worked in 8 different hospitals in Sweden, most of them in large cities (5 hospitals) and fewer in small towns (3 hospitals). 8 of the participants were female and 6 were male. The mean time of working in nephrology was 14.6 years (range 2–32 years). 10 were specialists in nephrology, of whom 9 were consultants, and 4 were nephrology residents who had not yet specialized. The participants received both written and oral information about the purpose of the study and about the clinical experience of the researcher conducting the interviews. While some worked in the same region as the interviewer, they did not work in the same clinic or unit.

The interview guide was developed by the research team and is shown in Table 1. It was based on a prior understanding of the challenges in the area, taking into account current regulations and guidelines on patient information. An external research group, with long experience in qualitative research in both nephrology and other clinical areas, was consulted about the interview guide and the method for data collection. The resulting guide consisted of pre-written questions. In this sense the interviews were structured, but follow-up questions were inserted, and participants were given the opportunity to express any thoughts they had on broader issues more indirectly related to the questions being asked. The questions were worded to encourage participants to share different experiences and thoughts about providing information to patients approaching the decision of whether to start dialysis, and which modality to choose. This included their practical experiences as well as their thoughts on how these conversations should ideally be conducted.

**Table 1:** Questions in the interview guide (originally in Swedish and translated to English by the author)

1. How long have you worked in nephrology?
2. How is the clinic where you work organized? How many doctors do you have? Do you have both an inpatient, dialysis and outpatient clinic?
3. How long have you been caring for patients with end-stage kidney disease who are facing the decision of long-term dialysis treatment?
4. There are many things to discuss with patients and their families when considering future dialysis. I imagine you have had these conversations. How are they usually conducted?
5. Do you have a plan for these conversations or are they more spontaneous? If you have a plan, what is it?
6. What do you think is important for these conversations to be good?
7. Do you think that there are any challenges to this type of conversation?
8. What do you think is important to say to patients for whom dialysis is a future option?
9. Are there things that patients ask about that you do not include unless you are asked?
10. Do you customize information for patients and their families, and if so, what might influence what you tell them or how you phrase the information? (For example, do you ever choose to phrase information in a way that you think will make it easier for the patient to make a *good* decision, according to your view? )
11. Have you had conversations with patients who have expressed in some way that they do not want to know so much about their illness or future treatment? How do you respond to this request?
12. As a patient or family member, what would you want to know before starting dialysis?
13. Several types of considerations may influence what treatment options one offers or recommends to patients (not just what one thinks will benefit the patient). Some of these may not be known to the patient, such as local routines or considerations of prioritization and resource allocation. Do you mention such considerations in your conversations with patients? (e.g., availability of hemodialysis, accessible dialysis modalities, practical details that guide treatment)

### Ethical considerations

Participants received written information about the study before agreeing to participate. When meeting the interviewer, they were again given written and oral information about the aim and procedures of the study, that participation was voluntary and that they had the right to withdraw at any time and to choose not to answer a question. They were informed that the interviews would be recorded, and that the data would be kept confidential. Written and oral (recorded) informed consent was obtained from all participants. This study involved no experiments. It was performed in accordance with relevant guidelines and regulations. Ethical approval was granted by the Swedish Ethical Review Authority (Dnr 2022-00652-01).

### Data collection

The data were collected between May and October 2022 through 14 semi-structured interviews, of which 4 were conducted on-site at the participant’s workplace, and 10 were conducted online. The decision to do the interviews on-site or online was mainly based on location of the interviewer in relation to the participant, and on the participant’s personal preferences. The interviews were conducted by one of the authors and not preceded by a pilot study. The interviews lasted on average 55 min (range 33–101 min), and 13 h in total. Immediately after each interview, the interviewer took brief notes on the main overall impression of the interview. The interviews were recorded and transcribed verbatim, then listened to a second time to correct and complete the first transcription. The transcriptions were made in close connection with each interview. Both recordings and transcriptions were pseudonymized and made available only to the authors of this paper. Based on the results from the 14 interviews, data saturation was achieved [10].

### Data analysis

The data were analyzed using qualitative content analysis in the broad sense elaborated by Graneheim et al. [11]. Content analysis was chosen as a suitable method to provide rich data about the nephrologists’ own experiences and reflections on these experiences. The interpretative approach was largely inductive, allowing the interviews to speak for themselves without being filtered through any particular theory. However, the identification of themes and other interpretative choices are inevitably influenced to some extent by background assumptions about the clinical and informational issues with which nephrologists might struggle. All authors read the transcriptions individually before initial discussions took place. Meaning units were identified and then condensed by the first author, who also coded them into categories and sorted them into major themes, representative of the patterns that emerged from the data. To ensure dependability during the process, the codes were kept in a descriptive mode, close to the transcription [12]. When identifying categories and themes, a process of abstraction and interpretation of the codes was carried out [11, 13]. Categories and themes were discussed several times within the research team until agreement was reached. They were compared with previously published findings to ensure the confirmability of the results [12]. The sub-themes are illustrated by selected quotes from the transcriptions, where appropriate, but due to space limitations, it was not possible to illustrate every finding under these sub-themes with a quote.

## Results

The participants shared both their experiences of actual conversations and their views on what the conversations ideally should be like. These perspectives could sometimes be voiced in the same sentence, which occasionally made it difficult to separate the participants’ characterizations of their actual practices from their normative views on the issues. The results are presented below, organized by the overarching themes and their sub-themes. They are also summarized in Table 2. Quotes from all the participants are presented, with a range of 1–6 quotes from each participant. The quotes are numbered consecutively in the order in which the interviews were conducted.

**Table 2:** Comprehensive view of the results sorted into themes and subthemes

Objectives	Content	Challenges	Strategies
Patients making their own decisions	Educating patients about the purpose and practical arrangements of dialysis	Sharing knowledge in a transparent but gentle way	Strategies to improve patient understanding and empowerment
Patients making a decision that is in their best interest	Informing patients about what life will be like on dialysis	Allocating time and timing conversations	Strategies to enable patients to make the “right” decision
	Informing patients about the influence of health care priorities	Dealing with other sources of information	Strategies to ensure social support from family
		Coping with the uncertain consequences of the decision	

### Objectives of information sharing

Two goals emerged as particularly important. Participants said they wanted patients to make their own decisions about the treatment, but the information should at the same time help patients decide what the nephrologist believes is in the patient’s best interest.

#### Patients making their own decisions

Several participants stressed that the decisions concern the patient’s own life and body, and that the patient therefore should decide whether to start dialysis and which modality to choose. The decision-making process was felt to be easier when the patients knew what they wanted.

*If they themselves know what they want*,* really know what they want*,* then it is*,* well*,* easier to deal with. (8)*

*I think that it’s wrong to put it like that*,* to let the physician…to leave the decision to the physician. I absolutely don’t want that. I want the patient to make her own informed decision*,* preferably based on my recommendation*,* but I accept just as well if the patient wants something else. (3)*

Making decisions early in the course of the disease was a goal that emerged in close relation to empowering patients to make their own decisions. One reason for preferring early decision making, as expressed by some participants, was that postponing the relevant decisions was seen as running the risk of patients losing some of their decision-making capacity and leaving the decision to someone else.

*It’s going to be a problem and it. or it can be a problem when you’re approaching dialysis and you haven’t really been able to talk about it and the patient is too ill or too tired and then... then it’s very hard for the patient to make a decision and then we... then it’s going to be the case that we take over the decision from the patient and we don’t want it that way. (6)*

*And it’s easier for them to get information now than when they are very ill. Because now they can influence choices and they can assimilate information in a better way. And they can also influence what health care does for them... much better*,* because otherwise someone else will decide for them. (5)*

#### Patients making a decision that is in their best interest

Some participants expressed that they feel a duty to inform patients in a way that helps them make choices that are in their own best interest, as judged by the nephrologist. If they are certain that one treatment option is superior, in terms of expected benefits and risk of harm, they want the patient to choose that option. The information that the patient can choose not to start dialysis was not always included when starting dialysis was considered the only reasonable option for the patient.

*I’ve noticed that when you push for PD [peritoneal dialysis] and so on*,* it can be like a pressure on them to choose that even if they don’t want to. Uh... And we think that we have good ambitions here to somehow preserve their residual function and from our professional point of view we say that it’s always a better quality of life. We think so*,* but it doesn’t have to be that way and the patient maybe almost*,* well*,* feels it and kind of feels forced and if they make a different decision*,* they’re kind of going against us*,* not pleasing us in some way*,* maybe. (7)*

*The professional role of the doctor is always to somehow evaluate which treatment might be best for the patient or which treatment might be less good*,* even if there are more choices*,* I think the way doctors work involves that. Then they might think that we are taking over a little bit too much*,* that... I’ve heard that. Not about me personally*,* but about doctors in general. (6)*

*But you often steer the patient*,* or try to steer them*,* toward what you think is best. (8)*

Promoting patient self-management was considered important in helping the patient to a greater quality of life, to more independence and freedom, and this objective was reflected in the information provided.

*If it’s not someone who we think is clearly incapable of managing their own dialysis*,* then maybe we push a little bit more for self-managed dialysis. We try*,* so to say*,* to explain to the patients that we think it’s the first choice*,* to manage your own dialysis. (9)*

*We prioritize self-management because we believe it’s better for the patient*,* and we know that many patients can do much more than they think. (10)*

### The content of the information provided to patients

Three themes were identified related to what content the information provided to the patient should have. The participants commented on information provided by all professions. While some information was expected to come from the nephrologist, much of the practical information (where to go, delivery of material at home or transport to the hospital, dialysis access planning etc.) was left to the specialized nurse.

#### Educating patients about the purpose and practical arrangements of dialysis

Several participants emphasized that important information includes the purpose of dialysis, how symptoms are treated, and the main differences between dialysis and conservative kidney management.

*It’s*,* well*,* it’s a big part of the information that’s about*,* well*,* what is kidney failure and what is dialysis (...) because we have to explain why you get dialysis. (6)*

*First of all*,* the patient should understand what it’s all about. I think so. Purely factual*,* both about the disease and the treatment and the future. (4)*

*In general*,* it’s important to tell the patient about the conservative way*,* that it’s a possibility and that... it could lead to a better quality of life*,* uh. Then you should also*,* well... there you should also evaluate if you think it’s really going to be like that. It’s... in some situations it would be unreasonable for a patient to choose that. (7)*

Some participants also emphasized the importance of communicating the fact that dialysis does not cure the disease, something which they said was a common belief among patients.

*Yes*,* but it’s not the case that I give the feeling that it’s just temporary or that you’re somehow cured by dialysis. In other words*,* it’s an adjunct to treatment when the kidneys stop working. And that’s what I tell them from the beginning: The kidney function that you’ve lost is lost*,* you can’t get it back. (3)*

#### Informing patients about what life will be like on dialysis

Most participants stressed the importance of providing an awareness of what life on dialysis will be like, including the limitations it imposes on the patient’s life, the fact that not all symptoms are relieved (e.g., fatigue), that dialysis is time consuming, and that it does not always improve the quality of life. The fact that life on dialysis also involves many restrictions on fluid and food intake was also considered important to communicate. Some participants mentioned that dialysis is much like a part-time job in terms of time commitment.

*You make the patient face a situation where they move from being very... well... maybe not that affected in their daily life by their kidney disease to a situation where they’re suddenly... that it’s something that’s going to largely dominate their whole life. So of course*,* I think for all patients*,* whether they’re prepared or not*,* when it’s finally time to start dialysis*,* it’s going to be a huge limitation on their life. (9)*

In contrast to this kind of negative information, some participants wanted to convey a sense of normality, of continuing to live one’s life, even with dialysis.

*Admittedly*,* you’re bound to this treatment a few days a week*,* but otherwise you can live a completely normal life and you can travel*,* because then we arrange the dialysis in that place (...) Life does not end once you start dialysis. You can actually live a completely normal life. (4)*

Addressing potential complications was considered essential, but not all complications were fully explained to patients, or they were downplayed, partly because the patients were not considered ready for this information and partly because complications do not always occur.

*What I think is important to inform patients about is that there’s a great risk that you will have to stay in the hospital for various reasons when you’re on dialysis. Because there will be complications (...) There will be a great disappointment regarding the whole health care if you don’t know it from the beginning but then you can’t say either: You will have to stay at the hospital so and so many times*,* because not all patients are affected in the same way. (7)*

*You should not completely take away the hope from those patients who are not candidates for transplantation*,* so clearly it becomes a little bit colored... well*,* that you cannot let it be*,* or let it... sound completely impossible to be on dialysis… but that you should still prepare them for the fact that there might be complications*,* but we take it one step at a time and for many it will be smooth. Something like that. (13)*

#### Informing patients about the influence of health care priorities

One interview question concerned whether information about health care priorities (e.g. due to resource constraints) is communicated to patients when such priorities underlie the nephrologist’s recommendations. There were conflicting views about the impact of resource constraints on decision-making, ranging from not being important at all, to reducing the frequency of dialysis, starting dialysis later, and encouraging self-management rather than staff-managed dialysis in the hospital.

*It’s not the case that we refuse patients to start dialysis. Sometimes it’s delayed because of a lack of resources (...) Resources can affect the start of dialysis*,* the choice of dialysis treatment*,* so these considerations are there in the background. (1)*

Many of the participants stated that patients should not be informed about the resource constraints that influence treatment recommendations, but that it is the nephrologist’s job and responsibility to take this into consideration.

*This is perhaps something that may not affect the individual patient (...) but it feels likewemust deal with it. And we must be aware of it*,* but we shouldn’t involve individual patients in it*,* this is our responsibility. (1)*

*But economic factors can influence the choice of treatment*,* but I (...) don’t think it really belongs here. I think there are many other important things for the patient to think about*,* so they should not have to face that. (6)*

Treatment options that could be explained (at least in part) by resource constraints were sometimes disguised as providing clinical benefit.

*We’ve had it before*,* when HD [hemodialysis] nurses kind of quit en masse and we were about to lose a whole dialysis unit*,* so there was panic*,* so it was just to... But I don’t think anybody said it was because we were understaffed*,* so to speak. We tried to say*,* “This is the best thing for you”. (2)*

*I don’t think we’re forced... force the patient into another dialysis modality because of resource constraints. But it could be that you emphasize what you think... the positive things that are still there. It must be medically motivated not to choose HD*,* so that... But maybe you have another conversation about PD and*,* yes... (7)*

### Challenges when informing patients and making decisions

Several challenges were addressed connected to discussing treatment options with patients. These included balancing honesty with being gentle, ensuring patient understanding, allocating time and timing conversations well. Dealing with competing sources of information, some of which were perceived as misleading, and coping with the uncertain consequences of decisions, were also challenges to the nephrologists interviewed.

#### Sharing knowledge in a transparent but gentle way

Some participants highlighted the importance of clear and transparent communication. However, some also acknowledged that transparency can be problematic if the information about treatment options, their likely effects and potential complications is negative, which may cause patients to feel sad, upset, anxious, or make them lose hope. They emphasized that honesty must be combined with sensitivity to the patient, so as not to cause unnecessary anxiety. Some participants said that they often feel the need to omit some of the more unfavorable information and to downplay the decisiveness of the decision at hand, expressing that decisions are not set in stone but can be changed, depending on the circumstances.

*And these are difficult questions for the patient*,* so sometimes... You could have talked to the patient and answered them*,* but their mind is somewhere else because this is a little bit threatening to life itself. So*,* it*,* it can be*,* yes*,* it’s about trying to dedramatize things and build trust. (10)*

*And not to worry the patients and so on... uh... clearly that’s an issue*,* but (sigh) if you deliver the information in*,* sort of*,* a kind way*,* then you could possibly avoid a lot of... uh... worry. (13)*

*Another thing that I think is important is that you can inform them that we are not locking ourselves into a certain form of treatment. I usually say “let’s try this and see how it goes*,* there are other treatment options”. (1)*

Clear and transparent communication was perceived as difficult when patients do not fully understand the situation they are in, and are unwilling or unable to receive information, making it impossible to share the same view on what the next step should be. Other related barriers identified by the participants were language differences, reduced cognitive function or low educational level, information overload or simply psychological inability to receive information, and belief in or resignation to fate. Some participants identified health illiteracy as the greatest challenge. Patients with limited ability to plan for the future and a perceived lack of proactivity or interest in their own care were also mentioned.

*The biggest challenge*,* as I said*,* is when you never... And it’s those patients who are... so to speak care illiterate*,* or how to put it*,* health illiterate*,* where you never get to a level where you can really talk about it (...) I think it’s more that maybe they’re not used to thinking... it’s kind of abstract thinking. There are a lot of patients who are not used to that. It requires... it requires quite a lot. It’s not about intelligence but it’s a kind of... a kind of ability that’s still required to be able to plan*,* to be able to... to be able to think about these things. (6)*

Understanding what it means to be on dialysis was identified as crucial for patients but still difficult to achieve.

*Because it’s going to highly affect the patient’s life later on and it’s really difficult for those who have never seen it or experienced it and... I think really or... yeah*,* I can imagine it’s hard to imagine what it’s going to be like. (14)*

#### Allocating time and timing conversations

The ideal relationship with the patient was seen as one that begins early in the course of the disease. This allows information to be shared at multiple points in time, enabling patients to make an informed decision about starting dialysis. According to some participants, this ideal is not always realized. Sometimes dialysis has to be started urgently and there is no time to discuss the decision in detail with the patient.

*I think that it’s good if you have some time to*,* well*,* repeat certain things and try to say things in different ways if it’s... if it can help*,* and that they have a chance to ask the questions that they have and so (...) It’s actually a very big decision to make (...) So I think sometimes it feels a little bit stressful... to have these conversations. (14)*

*It’s easier when you have more time*,* then you can also say to the patient*,* or that you say during the first visit: “We’ve said this*,* we can continue to talk about it when we meet again in six months. Think about it*,* so to speak*,* exchange thoughts with your family” (...) It’s more depth in it when you have time (...) but it’s... I find it more comfortable when it goes a little slower. That you get around... that they catch up. (8)*

Frustration could arise when patients were unable or unwilling to understand or adhere to a treatment plan, as this could interfere with a timely introduction to the chosen treatment option. Some patients were described as having unrealistic expectations of the future.

*They say “Yes*,* I understand what you are saying*,* that... that day will come*,* but we’ll leave it for then”. That “I don’t want to think about it right now”. And then they say*,* they very often add some kind of hope that*,* hope that this will not be necessary (...) And then they want to sort of push it away from themselves and hope that it… that the kidneys will just stay intact. (13)*

#### Dealing with other sources of information

There was a concern that the patients’ decision-making could be negatively influenced by competing sources of information, coming from different professionals (nurses, physicians), from other patients, and from sources outside the hospital, such as social media. There was also a concern that patients might get a false impression of certain dialysis therapies due to biased information.

*At the same time there is information that circulates between patients and those who have had a negative experience with something*,* they express it (...) And that discourages many patients from doing it because they don’t want to*,* they don’t know the details*,* but they still think that*,* then*,* it’s not a good method. (5)*

Similarly, ensuring that the family stays well informed can be challenging when patients inform the family in their own way, as this could lead to misunderstandings.

*It’s often the patient who talks to the family and then you notice that the information also gets... it’s*,* as I was about to say*,* a bit like Chinese whispers (...) I’ve experienced that you have an open dialogue with the patient*,* but the patient doesn’t have an open dialogue with her family. Something that we may not be able to do anything about*,* but that affects the treatment situation a lot. And you may not discover that until very late. Or maybe not at all. (1)*

#### Coping with the uncertain consequences of the decision

Many participants expressed that they want to recommend the best treatment for their patients, but they acknowledged that a lack of first-hand experience and a limited understanding of what it is like to live with dialysis makes it difficult to decide on behalf of their patients.

*Yes*,* we can be told about it*,* but I don’t think that… At the end of the day it’s probably still quite difficult to really have a full understanding of what it means for the patient. (9)*

Concerns were raised about decisions with unforeseen negative consequences and about starting the “right” patient at the “right” time, not too early and not too late.

*The difficulty is*,* well*,* if you start too early*,* it’s that you’re starting such an exhaustive treatment too early for a patient who could have waited and... you’re affecting their quality of life enormously*,* so to speak. Too late means going to the hospital for different complications*,* so to speak*,* that could have... been avoided with dialysis. (12)*

### Strategies for advancing goals and addressing challenges

The participants described a variety of strategies for advancing the goals and addressing the challenges described above, related to both patient empowerment and to more paternalistic goals, such as guiding patients to choose what the physician believes is in their best interest.

#### Strategies to improve patient understanding and empowerment

Some participants described that they try to create an appropriate setting for the conversation with the patient by adjusting the time and place of the meeting. This included adapting the amount and type of information the patient could process at the time and, where appropriate, spreading information over several appointments. Information could also be repeated to avoid misunderstandings. These strategies were used to prepare and educate patients over time.

*It may be that you don’t need to provide all the information at once. But maybe it’s the case that you have to portion it out a little bit. (1)*

*It’s really one of the purposes of following up with patients in the nephrology outpatient clinic*,* educating them about*,* uh*,* dialysis*,* preparing them mentally for what’s coming. (13)*

*But to share and to listen and to receive information*,* and it should be repeated several times*,* and it should be a joint decision. (11)*

The information was, according to some participants, tailored to suit the patient’s needs and abilities. The adjustment could be based on subtle impressions that arise during the conversation with the patient, and the ability to receive such impressions was believed to increase with professional and personal experience. Other relevant factors included the patient’s diagnosis, rate of progression, cognitive capacity, language, level of education as well as cultural factors.

*Some kind of interpersonal sense that you have*,* how... how you perceive this patient*,* if you think that... what words to use and what level to be on... how much information to give... eh... every time*,* so to speak*,* in one appointment. (12)*

Information was sometimes written down or illustrated to help patients understand. An interpreter was used to overcome language barriers between physician and patient. If they were unsure whether the patient understood the information, some participants said they asked the patient to repeat it. Sometimes, they encouraged the patient to bring a family member for support.

*If it’s a cognitive issue*,* a naturally aging brain*,* then maybe you need to speak slower or to write more things down*,* then I tend to do that. Also to repeat information more. And to encourage the patient to bring a family member to help and*,* yes*,* well*,* to receive the information and then to come up with questions. (8)*

That various professionals engaged in informing the patients was seen as valuable, but at the same time it was considered important that the information was framed in the same way by the different professionals.

*Because one*,* you put together a puzzle. And it becomes more complete when you get aspects from different angles and so on. (8)*

*You take turns... you have the nephrology coordinator or nurse*,* and you have to agree on how to phrase it. It’s almost like you must use the same words*,* the same expressions*,* otherwise... the patient gets lost. (5)*

#### Strategies to enable patients to make the “right” decision

Many participants said that it is common practice to frame information about the pros and cons of different dialysis treatments, or of not starting dialysis, in such a way that the patient’s decision is guided by what the physician believes is the best option for the patient (referred to here as the “right” decision), perhaps without explicitly recommending which option to choose.

*Sometimes we actually have a preconceived notion that this is the best treatment for this patient based on our long experience and so on. Then I can sometimes feel that we are pushing the patient a little bit with our language towards a certain choice or a certain treatment or... uh... for better or for worse. We think we are doing good*,* and that might be based on factors that we think we have seen and what would be medically appropriate. (1)*

*I’m not saying you slant the information*,* but you... want to present what you think is good with a certain method*,* and it probably gets a little colored in this case. (7)*

*But of course*,* we emphasize the positive parts (laughter) and we… we kind of paint a positive picture of the future. If I have somebody who I think... here it will be more conservative management*,* then we try to talk more about the negative parts of dialysis*,* maybe. (11)*

*But then it’s mainly for patients where I think pretty clearly that this could be a good treatment option for them. Then I give them more information about dialysis (laughter). If I see early on that it’s a patient with cognitive difficulties or a severe comorbidity of some other kind where I don’t think it’s a good treatment option*,* then I can mention that*,* but I can also talk about what you can... what treatment you can get if you don’t get dialysis. (14)*

The information could also be framed with the intention of forcing the patient to make up their minds (rather than make a specific decision), for example by emphasizing the rate of progression of kidney failure to convey the urgency of the decision to be made.

Some participants said that there were cases where they decided for the patient, because they felt it was the most appropriate solution due to the complexity of the decision or the situation. The idea was that patients lack experience of living with dialysis, making it almost impossible for them to make a truly informed decision.

*I often find that it’s really difficult for the patient if they don’t really... if they don’t have any previous experience. (6)*

*Some say “But I can’t make any decision. What do you think I should do?” And that’s actually okay. Uh... because they’re facing something they’ve never experienced before*,* so it’s... they don’t have enough information. They will never have enough information until they have tried both methods. So*,* I understand that. It’s... then I help them. Then I decide for them*,* and then they are satisfied. (5)*

#### Strategies to ensure social support from family

The participants emphasized the importance of having family members present during conversations, to prepare them for the role they are expected to play and the responsibility they will (have to) assume. The disease and its treatment affect the relationship, work, and the ability to do things in everyday life (keeping pets, going to a summer house, bathing, traveling). Family members may also be affected by changes in the patient’s physical appearance due to weight loss or gain, catheters for dialysis, etc., and the nephrologists considered it important that family knows this in advance so that family members can support the patients and not leave them in the lurch.

*We have to put all the cards on the table and give them the opportunity to decide how we’re going to deal with this together and... It’s important that family members are present and informed about what this means. (3)*

## Discussion

Early assessment and planning for patients with end-stage kidney disease, as preferred by the nephrologists we interviewed, is strongly recommended by the global nephrology community [14]. The ideal is to reach common ground in terms of shared knowledge, shared views on the appropriate decision-making process, and ultimately agreement on which treatment option is the best. There is a push to recognize patient-centered goals and preferences in nephrology and to translate this into practice, while recognizing the challenges [9, 15–17]. This study further addresses the challenges that nephrologists face in preparing patients on the path to end-stage kidney disease and the decision about future dialysis.

Nephrologists may find it difficult to navigate between competing goals when sharing information with patients. The participants in our study emphasized that patients should ultimately decide whether to start dialysis or choose conservative kidney management, since it is their life and well-being that are at stake, and that information should serve to facilitate self-determination and to help patients take responsibility for their own treatment. At the same time, the interviews also reflected a more traditional paternalistic perspective. Information could be framed in a way that encourages decisions that make the most sense to the nephrologists and also avoids making patients feel bad about the situation. This may not only undermine patient autonomy but can also lead to disappointment when patients experience the reality of life on dialysis [18]. This tension between promoting patient autonomy and paternalism has been demonstrated in previous research [19, 20].

When nephrologists decide which options to put on the table and how to frame them, they may not only have the individual patient’s best interests at heart. Prioritization considerations may result in the physician recommending a particular treatment option, delaying treatment, or offering a less effective treatment to the patient. Such considerations are not always communicated to patients [21, 22], which is consistent with the findings of our study. The nephrologists in our study did not consider such information important or considered it too burdensome for the patient. However, it could be argued that patients should be made aware of prioritization considerations, even if they have little use for this information in their decision-making [23].

Nephrologists may occasionally seize control to actually respect patient self-determination. Participants in our study described that patients sometimes want them to make decisions and take full responsibility for those decisions. The variability in patient preferences regarding the level of involvement in clinical decision-making has been noted in previous studies [24]. Respecting patients’ wishes not to receive certain information is, of course, in a sense respecting patient autonomy, but it may also result in patients giving up meaningful participation in the process [25]. This tension serves as a reminder of the importance of a continued discussion about different, potentially conflicting, notions and ideals of autonomy. Postponing decisions to allow for patients to feel more ready to discuss and make decisions could be seen as a practical solution, but it carries the risk of closing the window of opportunity for patients to participate in these decisions, and especially so for this group of patients with a chronically deteriorating disease [26].

Many of the nephrologists’ considerations regarding what information to share, how to share it and when to share it seemed to be based on certain assumptions about patients. For example, the inclination to stress certain facts about dialysis and life on dialysis appears to be based on a perceived need to correct or prevent certain expectations. Likewise, the timing of information efforts and the particular ways in which the participants thought they should frame things to facilitate understanding were based on certain assumptions about how participants could process information, what they might find burdensome, etc. The extent to which these assumptions were based on knowledge of individual patients, previous interactions with patients, or something else is not clear. More research is needed on this topic.

Part of the complexity of informing the patients, our findings suggest, is related to uncertainty. Not only is there often clinical uncertainty; it is the patient who will experience dialysis, something that the physician may never do, making it difficult for the latter to describe with full confidence what it will be like. At the same time nephrologists may feel the need to assume the responsibility that comes with the inevitable information advantage of knowing much more about the *medical* complexity of end-stage kidney disease, the mechanisms of dialysis treatment, and the effects of various medications. Mutual recognition of the limitations of the nephrologist’s and the patient’s respective areas of knowledge will be key to making the right decisions in this clinical setting.

Many of the information-related challenges that the participants faced are similar to those described in other studies. For example, previous research has shown that differences in language, age, ethnic background, education level, health literacy, and cultural barriers can negatively impact chronic kidney disease patients’ participation in care due to both lack of ability and motivation to participate, which is consistent with the findings of this study [15, 27–29]. Previous research also shows that patients do not always receive information tailored to their own needs and preferences [19, 30]. Nephrologists in our study attempted to overcome barriers to patient understanding through, among other things, repeated communication, information from multiple sources, and appropriate timing. Despite these efforts, a common understanding was not always achieved. Family members and other patients are seen as a resource in the information process but can also hinder patient understanding and decision-making. This has also been shown in previous studies [30–32]. Individualized information and decision support are needed for both the patient and the family [33, 34].

A strength of this study is that the participants came from different hospitals, located in both cities and smaller towns in Sweden. They had various experiences, making the data rich. As for possible limitations, it should be noted that the transferability of the findings to other countries or settings might be affected by the fact that the participants only work in Sweden. In addition, the particular questions asked could steer the participants in a direction chosen by the researcher. However, the use of supplementary questions to clarify and expand on certain topics that seemed important to the participants reduces this risk. Content analysis, broadly defined, was chosen as an appropriate method for exploring nephrologists’ own experiences and reflections on these experiences, and our approach to interpretation was largely inductive. By ensuring that the interpretation of the data was close to the wording in the transcriptions, we aimed to minimize the risk that the results would be biased by the researchers’ prior understanding of the topic. The interpretation and abstraction of the data was also done with the co-authors, who are not clinicians, to limit the influence of the experience in the field of one of the researchers, who is a nephrologist.

Inviting nephrologists to participate through an email list carries the risk of self-selection bias. For example, the participants may have a greater interest than other nephrologists in issues related to information sharing and conversations with patients. However, it was our assessment that other feasible recruitment methods would involve a similar risk of self-selection. The small sample size would obviously be problematic if one were to aim for results generalizable to the global population of nephrologists. That was not, however, the aim of this study. Data saturation was achieved, indicating that the study objective was met with respect to the specific study population. Further research with a more diverse and systematically selected population of participants could complete the picture.

## Conclusions

Treatment planning for patients with chronic kidney disease requires a common understanding between the patient and the nephrologist of the disease status and treatment options, the impact of the disease and treatment on the patient’s life, and the patient’s preferences and priorities. There are several barriers to achieving this common understanding. In this study, we found a tension between the desire to help patients make a decision that is in their best interest, as perceived by the nephrologist, and the value of encouraging patients to make their own decisions. Nephrologists expressed a sense of professional responsibility to make good treatment recommendations for their patients, and to adapt to patients’ needs and preferences, which was seen as difficult due to a lack of experience of what life on dialysis is like. Balancing the need to be transparent about what life on dialysis *might* be like with the need to be gentle and reassuring to the patient and not burden the patient with negative or unwarranted information, was also seen as a challenge. Navigating competing sources of information, timing information efforts well, and repeating information, emerged as important to ensure patient understanding and empowerment, something which was not always possible due to patients’ lack of capacity or desire to postpone or delegate decisions. These results may inspire future research and have important implications for the further development of support for nephrologists in sharing information with patients as they progress towards end-stage kidney disease.

## Data Availability

The dataset cannot be made publicly available for ethical and legal reasons. Access to the data can be granted on reasonable request. Requests to access the data should be sent to: registrator@lu.se.
